# Printed Thick Film Resistance Temperature Detector for Real-Time Tube Furnace Temperature Monitoring

**DOI:** 10.3390/s24102999

**Published:** 2024-05-09

**Authors:** Zhenyin Hai, Zhixuan Su, Kaibo Zhu, Yue Pan, Suying Luo

**Affiliations:** Department of Mechanical and Electrical Engineering, School of Aerospace Engineering, Xiamen University, Xiamen 361005, China; 19920221151557@stu.xmu.edu.cn (Z.S.); 34520222201222@stu.xmu.edu.cn (K.Z.); 34520222201055@stu.xmu.edu.cn (Y.P.); 34520222201040@stu.xmu.edu.cn (S.L.)

**Keywords:** tube furnace, thick film, silver, high-temperature, blade-coating

## Abstract

Accurately acquiring crucial data on tube furnaces and real-time temperature monitoring of different temperature zones is vital for material synthesis technology in production. However, it is difficult to achieve real-time monitoring of the temperature field of tube furnaces with existing technology. Here, we proposed a method to fabricate silver (Ag) resistance temperature detectors (RTDs) based on a blade-coating process directly on the surface of a quartz ring, which enables precise positioning and real-time temperature monitoring of tube furnaces within 100–600 °C range. The Ag RTDs exhibited outstanding electrical properties, featuring a temperature coefficient of resistance (TCR) of 2854 ppm/°C, an accuracy of 1.8% FS (full scale), and a resistance drift rate of 0.05%/h over 6 h at 600 °C. These features ensured accurate and stable temperature measurement at high temperatures. For demonstration purposes, an array comprising four Ag RTDs was installed in a tube furnace. The measured average temperature gradient in the central region of the tube furnace was 5.7 °C/mm. Furthermore, successful real-time monitoring of temperature during the alloy sintering process revealed approximately a 20-fold difference in resistivity for silver-palladium alloys sintered at various positions within the tubular furnace. The proposed strategy offers a promising approach for real-time temperature monitoring of tube furnaces.

## 1. Introduction

There is a wide range of temperature testing needs for pipeline structural components in the fields of energy and power, iron and steel metallurgy, and materials synthesis [1,2]. Tube furnaces play a pivotal role in material synthesis, serving as widely utilized heating apparatuses for high-temperature processing, heat treatment, and chemical reactions [3,4]. Variations in furnace temperature significantly influence material properties and crystal growth [5]. Hence, precise temperature measurement in a tube furnace is imperative for the material synthesis process. In addition, the tubular furnace needs to be calibrated regularly to ensure the uniformity and accuracy of the temperature in the furnace [6]. Conventional methods for periodic maintenance of tube furnaces include temperature measurement using armored thermocouples and temperature blocks [7]. Although commercially armored temperature sensors are capable of withstanding higher test temperatures, they often face challenges in installation and fail to achieve the required testing accuracy [8]. The temperature measurement block assesses the temperature by the change of high temperature on its volume; this method cannot realize the real-time monitoring of the temperature of the tube furnace. Commonly employed non-contact temperature measurement methods include infrared temperature measurement [9], pyrometer temperature measurement [10], and others. Although the non-contact temperature measurement method has the advantages of testing safety and no pollution, there are still some disadvantages [11,12]. For example, infrared temperature measurement cannot monitor the furnace temperature in real time, making it unsuitable for such monitoring purposes [13]. Additionally, pyrometer temperature measurement is constrained by environmental limitations, making it challenging to achieve accurate temperature monitoring in dusty environments [14]. Levendis et al. developed a three-color optical pyrometer for temperature testing of tube furnaces, but it requires the furnace to be transparent and is difficult to apply to tube furnaces in dusty environments such as industrial production [15]. Contact temperature measurement methods, such as mercury thermometers and discrete-type sensors, offer advantages over non-contact methods for testing temperatures in tube furnaces, but they also present certain drawbacks [16,17]. Mercury thermometers are presently used at lower temperatures and are not suitable for temperature testing in high-temperature environments [18]. Temperature sensors based on semiconductor structures made of silicon and/or silicon carbide feature a wide temperature measurement range (20–600 °C), ultra-high sensitivity (−7786 ± 71 ppm/°C), and high precision characteristics. However, the utilization of surface micro-machining techniques presents challenges in fabricating sensor elements on large, curved substrates [19,20,21]. Patch-type temperature sensors are constrained by the limitations of high-temperature adhesives, limiting their use to lower temperatures and frequently encountering challenges in matching the stress of high-temperature adhesive [22]. Liu et al. developed an ITO/In_2_O_3_ thick film temperature sensor on a ceramic fiber felt substrate, enabling temperature monitoring from liquid nitrogen to 1200 °C. Nonetheless, it frequently encounters thermal stress matching issues under the influence of high-temperature adhesive, resulting in diminished sensor accuracy [23].

Recently, thick film temperature sensors enabled in situ temperature monitoring and offer advantages such as no damage to the substrate and low interference [24,25]. Techniques employed in the fabrication of thick film temperature sensors include magnetron sputtering [26], electro-hydrodynamic printing technology [27], and screen printing [28], among others. Although the magnetron sputtering process produces films of superior quality, it cannot deposit films on large structures due to the size limitations of the processing chamber and requires a long preparation cycle and high cost [29,30]. Electro-hydrodynamic printing technology enables the deposition of narrow linewidth thin/thick films with high precision. He et al. demonstrated this by using electrohydrodynamic printing technology to prepare nanosilver temperature sensors [31]. However, the range of ink viscosity that could be used in this technology was limited, and the temperature resistance of the sensor was only 105 °C. The blade-coating process is not only cost-effective and has a brief preparation cycle [32] but also allows printing on cylindrical surface substrates with large curvatures. For example, Zhao et al. demonstrated a platinum-platinum/rhodium thick film thermocouple using blade coating technology, showcasing the method’s capability to print on curved surfaces [33]. Currently, there are no studies related to the preparation of thin/thick film temperature sensors for real-time monitoring of tube furnace temperatures using the blade-coating process.

Here, we proposed a strategy where silver (Ag) resistance temperature detectors (RTDs) were fabricated using blade-coating on quartz ring substrates, creating temperature measurement rings for real-time monitoring of tube furnace temperatures. This strategy exhibited high scalability, accommodating an arbitrary number of sensors for monitoring tube furnace array temperatures, facilitating quick maintenance changeovers, and being expandable to monitor the health of other tubular components. Systematic studies were conducted on the microstructures of both the surface and cross-section of Ag RTDs, and the interfacial bonding strength between the Ag film and substrate was measured. The fabricated RTDs were calibrated and adjusted within the range of 100–600 °C, with their temperature coefficient of resistance (TCR), accuracy, stability, hysteresis, and response time measured. Furthermore, the consistency between the initial resistance and TCR of multiple RTDs was evaluated, and measurement errors induced by the fabrication process were reduced through calibration. Finally, the prepared temperature measuring rings were installed on the tube furnace for simulated isothermal annealing, cyclic annealing, and silver-palladium sintering quality assessment tests.

## 2. Materials and Methods

### 2.1. Experimental Materials

Ag paste (01H-1803) and AgPd paste (02H-2010D) were sourced from Shenzhen Sryeo Electronic Paste Co., Ltd. (Shenzhen, China). The utilized Ag paste contains 3–6 wt% of glass powder, primarily composed of alumina (Al_2_O_3_) and silicon dioxide (SiO_2_). Quartz ring substrates procured from Ate Quartz Preparation Co., Ltd. (Jiangsu, China). had an inner diameter of 50.5 mm, an outer diameter of 55 mm, and a width of 7 mm. High-temperature PET adhesive tapes with a thickness of 20 μm for the tape mask were obtained from Wo Sheng Tape Co., Ltd. (Hangzhou, China). An infrared laser was used to process a corresponding mask pattern on the tape, enabling the tape to serve as a flexible mask plate. The commercial Pt leads with a diameter of 0.2 mm and a nominal purity exceeding 99.99 wt% were used in this study.

### 2.2. Real-Time Monitoring Strategy for the Tube Furnace

**Figure 1:**
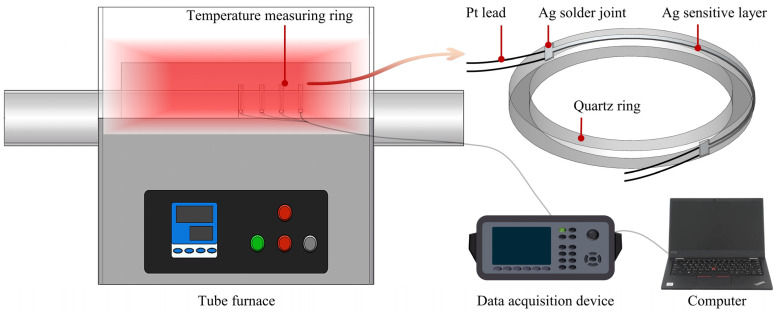
Schematic diagram of using temperature measuring rings for real-time temperature monitoring in a tube furnace.

A strategy was developed for monitoring tube furnace temperatures using Ag RTDs. As part of this approach, the Ag RTD was fabricated in situ on the quartz ring surface to form the temperature measurement ring unit. Figure 1 illustrates a schematic diagram of real-time monitoring of the temperature field in a tubular furnace using multiple temperature measurement rings. The tube furnace (OTF-1200X), procured from Hefei Kejing Materials Technology Co., Ltd. (Hefei, China), measures 1270 × 400 × 540 mm^3^ when closed. It is designed with an outer diameter of 50 mm, allowing for the installation of temperature measuring rings to measure temperature on the tube. Each ring is composed of an Ag-sensitive layer, Ag solder joints, Pt leads, and a quartz ring substrate. Ag was selected for the sensitive layer due to its high conductivity (3.13 μΩ·cm), high sensitivity (≥2340 ppm/°C), and high-temperature resistance (≥600 °C), enabling temperature detection through resistance changes [26,34,35]. Compared to precious metals such as Pt, Ag thick films are more cost-effective and can be deposited using a low-cost blade-coating technique. Based on this technique, the limitations of conventional magnetron sputtering, which struggles to deposit large-area patterns on curved surfaces, are effectively surmounted. Additionally, compared to thermocouples, RTDs generally provide higher accuracy at mid to low temperatures and do not require cold-junction compensation.

To mitigate the impact of contact resistance from solder joints and leads on temperature measurement accuracy, a four-wire resistance measurement method was employed. Ag RTD data were collected using a data acquisition card (KEYSIGHT 34972A (Santa Rosa, CA, USA)) and a computer. Multiple sets of temperature measurement rings were employed for synchronous real-time temperature monitoring at various positions within the tube furnace. This configuration provides a cost-effective solution to the challenge of achieving real-time multi-point temperature measurement in tube furnaces.

### 2.3. Preparation Process of Ag Thick Film RTD

**Figure 2:**
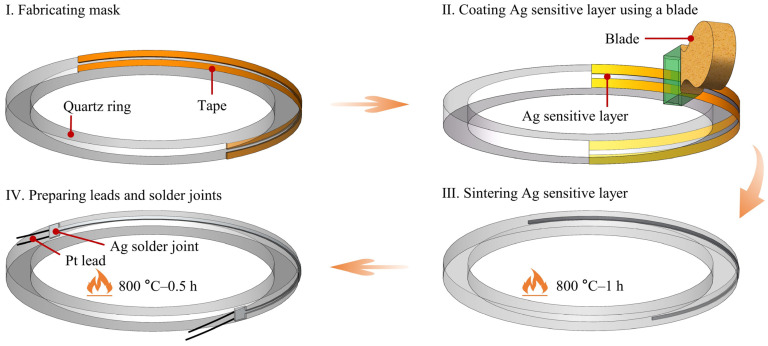
Fabrication process diagram of the Ag RTD.

Figure 2 depicts the fabrication process of the Ag RTD on a temperature measurement ring. Initially, the quartz ring substrate was sequentially ultrasonically cleaned with acetone, alcohol, and deionized water to remove dust and organic residues prior to RTD preparation. Afterward, the cleaned quartz ring substrate was dried using nitrogen gas to ensure the complete removal of moisture. In Step I, PET tape was affixed to the outer surface of the substrate, acting as a flexible mask for patterning the sensitive layer. The length and width of the hollow part of the tape were approximately 60 mm and 0.45 mm, respectively. A longer length of the sensitive layer results in a more accurate determination of the average temperature within the same temperature zone of the tube furnace. In step II, the ends of the quartz ring were secured using a fixture, followed by the preparation of the Ag-sensitive layer by a blade coating method. During this coating process, the blade was maintained at an angle of approximately 45 degrees relative to the substrate. The speed of application was maintained at about 10 mm/s, and the force exerted was approximately 0.3 N/mm^2^. In step III, the mask was removed, and the device underwent an annealing process at 800 °C, with a controlled ramp rate of 5 °C/min. Maintained for 1 h, this temperature facilitated the evaporation of organic solvents within the Ag-sensitive layer and promoted the coalescence of Ag particles. In step IV, Ag paste was used as the soldering agent to achieve electrical interconnection between the Pt lead and the Ag-sensitive layer. After initially fixing the Pt lead, the device was annealed again at 800 °C for 0.5 h to ensure a robust connection between the lead and the sensitive layer.

### 2.4. Experimental Setup

During calibration and validation, the Ag RTDs were placed in the constant temperature zone of the tube furnace alongside a commercial K-thermocouple (KPS-IN600-K-3.0, Zhongtou Trading Co., Ltd., Taizhou, China) positioned at the same location for temperature monitoring. Throughout the testing period, the tube furnace maintained heating and cooling rates at 5 °C/min. The unit of TCR, expressed in ppm/°C, indicates the relationship between the resistance of the RTD and the temperature, and it can be calculated as follows [36]:

(1) $$TCR=\frac{1}{R(T_{0})}\times \frac{R(T)-R(T_{0})}{T-T_{0}}=\frac{k}{T-T_{0}}$$

Here, *R*(*T*_0_) is the resistance of the RTD at the initial temperature, and *R*(*T*) is the resistance at temperature *T*. The slope of the fitted straight line is represented by *k*. The RTD’s stability at high temperatures can be assessed through the resistance drift rate (DR), expressed by the following Equation [37]:

(2) $$DR=\frac{\Delta R}{R_{ref}}\times \frac{1}{\Delta t}$$

where, at the constant temperature point, *R_ref_* and Δ*R* are, respectively, the initial resistance and the value of change in resistance of the RTD, and Δ*t* is the maintained time.

### 2.5. Characterization Techniques

The morphologies of the obtained samples were characterized by scanning electron microscopy (SEM, Zeiss GeminiSEM 500 (Jena, Germany)) combined with energy-dispersive spectroscopy (EDS). The adhesion strength between the Ag film and the substrate was measured using a scratch tester (WS-2005 (Lanzhou, China)) with a maximum load of 80 N and a scratch length of 4 mm.

## 3. Results and Discussion

### 3.1. Characterisation of the Ag RTD

**Figure 3:**
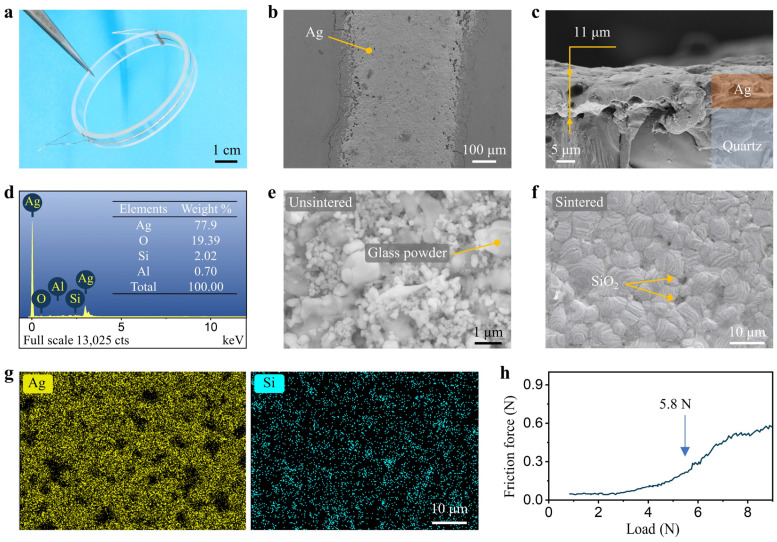
(**a**) Optical image of the Ag RTD on a quartz ring substrate; (**b**) SEM image of the Ag-sensitive layer; (**c**) SEM image of cross-section of the Ag RTD; (**d**) EDS spectrum analysis of particles in the Ag-sensitive layer; (**e**) SEM image of an unsintered Ag-sensitive layer; (**f**) SEM image at high magnification of the sintered Ag-sensitive layer; (**g**) EDS mapping images of the Ag-sensitive layer corresponding to (**f**); (**h**) Scratch test curve for the Ag sensitive layer.

Figure 3a displays the optical image of the Ag RTD, demonstrating that the sensor was successfully fabricated on a large, curved quartz ring based on the screen printing process. Figure 3b presents the surface SEM image of the Ag-sensitive layer deposited on the quartz substrate. The Ag-sensitive layer, despite a small number of holes, exhibits no apparent breaks on the surface or within the conductive network. The microstructure of the cross-section of the Ag RTD was characterized, further revealing that the sintered Ag film remained continuous and did not detach from the substrate, as shown in Figure 3c. These results indicate a good thermal compatibility between the Ag film and the substrate. Notably, measurements based on the scale of the image indicated that the thickness of the Ag-sensitive layer was approximately 11 μm, meeting the requirements for minimizing disturbances in temperature monitoring. Appropriate film thickness can enhance the TCR of thick-film RTDs, but excessively thick film thickness may lead to excessive sintering stress [38], resulting in film delamination or even substrate cracking [39]. Figure 3d depicts the EDS spectrum analysis of particles within the Ag-sensitive layer. Quantitative elemental analysis indicates that the main elements present were Ag, O, Si, and Al. The small presence of elemental O-element can be attributed to the passivation layer on the Ag surface at elevated temperatures and the presence of oxides in the glass powder [40]. The Ag film also contains trace amounts of Si and Al, originating from the glass powder in the Ag paste. To assess the morphological changes of the Ag-sensitive layer induced by the sintering process, SEM images of the film before and after sintering were analyzed. Before sintering, the nanoscale Ag particles within the sensitive layer exhibited a spherical morphology, accompanied by micron-scale glass powder observed internally, as illustrated in Figure 3e. Following sintering, the Ag particles within the sensitive layer agglomerated and formed numerous sintered necks, as shown in Figure 3f. Additionally, the glass powder was observed to have completely softened into an amorphous glass phase. EDS mapping images revealed that the elements of the glass phase were distributed at grain boundaries and on the surface of the particles (Figure 3g). Incorporating a small quantity of glass powder enhances interparticle contact conductivity and bonding strength, thereby mitigating the migration tendencies of Ag particles and improving the Ag-sensitive layer’s high-temperature performance [41]. The adhesion strength of the Ag-sensitive layer on a quartz substrate, determined using the scratch method, was approximately 5.8 N (Figure 3h). This value represents a doubling in adhesion strength compared to platinum on alumina substrate (2.9 N) [38]. The robust adhesion ensures the reliability of the Ag-sensitive layer under high-temperature conditions.

### 3.2. Calibration and Verification of Ag RTDs

**Figure 4:**
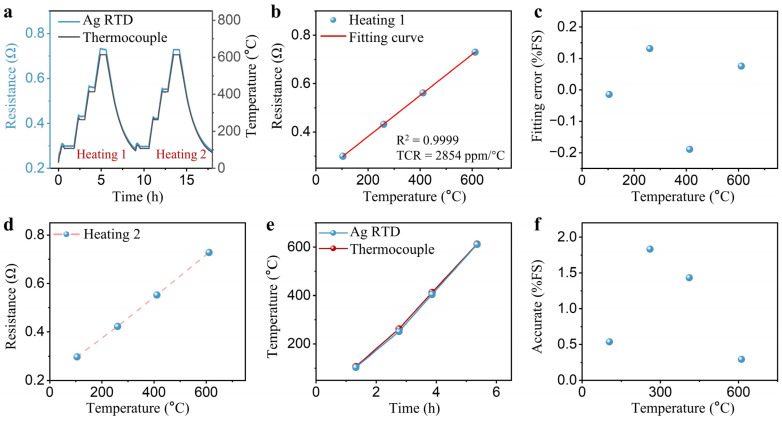
(**a**) Curves for RTD and thermocouple calibration and accuracy testing; (**b**) temperature-resistance fitting curve during the first heating of the RTD; (**c**) fitting error of the heating curve of the RTD; (**d**) temperature-resistance fitting curve during the second heating of the RTD; (**e**) temperature curves of the RTD and K-thermocouple; (**f**) accuracy of the RTD from 100 to 600 °C.

The accuracy and sensitivity of an RTD directly reflect its temperature monitoring capability. Figure 4a details the comprehensive calibration process for RTDs. Static calibration was performed on the RTDs, maintaining temperatures at 105, 261, 412, and 612 °C, with a temperature variation of less than 2 °C over a 10-min period, which ensured that the Ag RTD resistance values were under steady state at that temperature. In this stabilization phase, the output resistance of the RTD and the temperatures from the standard thermocouple were averaged for static calibration. The same protocol was employed for validation tests. Figure 4b shows that the calibration results for the Ag RTD demonstrated a goodness of fit R^2^ exceeding 0.9999 and a TCR of 2854 ppm/°C, closely matching the TCR of sputtered Ag film [26]. Furthermore, to quantitatively evaluate the fit’s impact on accuracy, residual errors at various temperatures were examined. Within the 100–600 °C range, the maximum fitting error for the Ag RTD was less than 0.19% of the full-scale (FS) error (Figure 4c). The high goodness of fit and small fitting error indicates that the fitted data were in good agreement with the experimental data, contributing to the Ag RTD’s accuracy. Figure 4d presents the results from the second calibration test round, showing electrical signal outputs closely aligned with those from the initial calibration across different temperatures. In the testing phase, the Ag RTD was compared with commercial K-thermocouples to verify temperature measurement accuracy (Figure 4e). As depicted in Figure 4f, temperatures measured by Ag RTDs and thermocouples reveal a maximum error of only 1.8% FS across the tested temperature range. These findings indicate that the RTDs demonstrated high accuracy throughout the test phases, facilitating precise temperature monitoring of tube furnaces.

**Figure 5:**
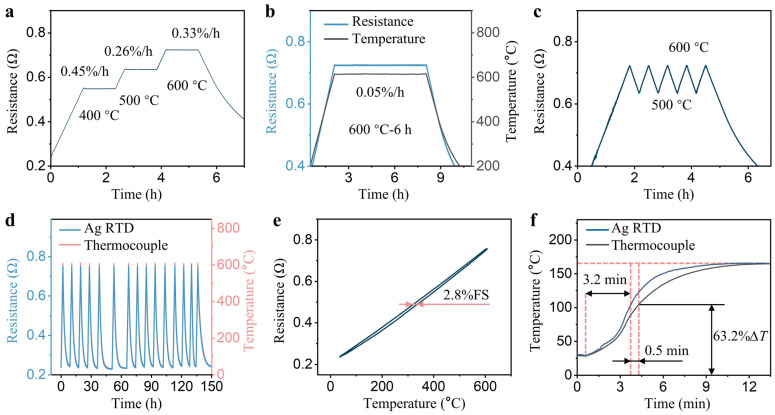
(**a**) Temperature drift curve of the RTD at 400, 500, and 600 °C for 1 h; (**b**) Resistance drift curve of the RTD at 600 °C for 6 h; (**c**) Five experimental cycles of the RTD between 500 and 600 °C; (**d**) Resistance changes curve of the RTD under 15 cycles; (**e**) Heating and cooling curve of the RTD; (**f**) RTD and armored thermocouple response times.

The stability of the sensor is a crucial parameter for evaluating the performance of RTD. Figure 5a demonstrates the Ag RTD’s high-temperature resistance stability at 400, 500, and 600 °C in an air environment, with a DR of 0.33%/h at 600 °C. To substantiate the long-term high-temperature stability of the prepared RTD, it was tested at 600 °C for 6 h (Figure 5b). The RTDs’ exceptional stability, indicated by a DR of 0.05%/h over a 6-h period, enables extended-duration, real-time monitoring of high-temperature tube furnace environments. To examine the RTD’s dynamic stability, additional tests involved heating and cooling cycles at 500–600 °C. Four cycles were conducted, revealing consistent temperature fluctuations and a maximum resistance growth rate of 0.31%/h (Figure 5c). Thermal cycling tests were conducted to evaluate the long-term performance and aging characteristics of the developed RTD. The RTD underwent 15 cycles from room temperature to 600 °C over a duration of up to 150 h (Figure 5d). The relative deviation between the peak resistance values observed in the 1st and 15th cycles was measured to be 0.36%, indicating that the RTD exhibits minimal degradation during long-term aging tests. A single round of ramp-up and ramp-down cycles, with rates set at 2.5 °C/min, was conducted to assess the inconsistency between the forward and reverse outputs of the RTD. The hysteresis error of the RTD was approximately 2.8% FS, as depicted in Figure 5e. The response times of the prepared RTD and commercially available armored thermocouples were compared in a tube furnace. The RTD showed a response time of about 3.2 min, while the thermocouple took about 3.7 min to respond, both from 28 to 165 °C (Figure 5f). The slower response of the thermocouple was attributed to its wire contact with the furnace tube and its larger diameter after armoring (3 mm). The prepared RTD’s average detectable heating rate exceeded the maximum recommended rate for the tube furnace, which is typically 10 °C/min, with the RTD reaching 10.7 °C/min.

**Figure 6:**
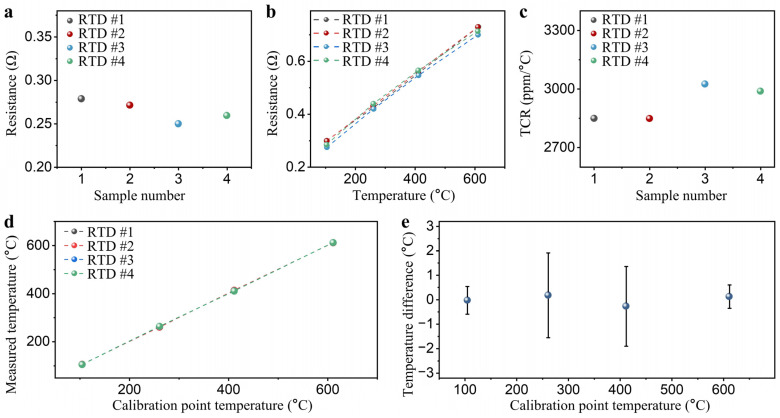
(**a**) Initial resistances of four Ag RTDs; (**b**) resistance temperature curves for four RTDs at 100 to 600 °C; (**c**) TCRs for four RTDs at 100 to 600 °C; (**d**) Resistance temperature curves of four RTDs after calibration; (**e**) temperature differences between the four RTDs after calibration.

The consistency of the initial resistance and TCR of multiple Ag RTDs prepared by the screen printing process was evaluated. Figure 6a shows the initial resistance of four RTDs at room temperature, with an average resistance of 0.265 ± 0.013 Ω. Figure 6b presents the heating curves of four RTDs, showing resistance increases with temperature rise but observing certain deviations among the temperature-resistance curves. In addition, the sensitivity of the four RTDs was calculated with TCR statistics ranging from 2847.4 to 3024.8 ppm/°C with a maximum deviation of 6% (Figure 6c). Although screen printing offers the advantages of a simple process, low equipment, and mask cost for preparing Ag RTDs, achieving uniform thickness and consistent performance of sensitive films on curved surfaces remains challenging [42]. Therefore, calibration of each RTD individually is required when multiple RTDs are used simultaneously to minimize measurement errors induced by the fabrication process [43]. During calibration, the relationship between resistance and temperature was determined by the initial resistance value and the TCR of the RTD. This enabled the conversion of measured resistance into temperatures. Following calibration, the measured temperatures of the four RTDs at the four calibration points, as shown in Figure 6d, exhibited excellent uniformity. Subsequent calculations revealed that the temperature difference of the four RTDs at the calibration points were all below 2 °C, laying a solid foundation for subsequent temperature field measurement applications (Figure 6e).

### 3.3. Application of Ag RTDs in Temperature Monitoring of Tube Furnace

**Figure 7:**
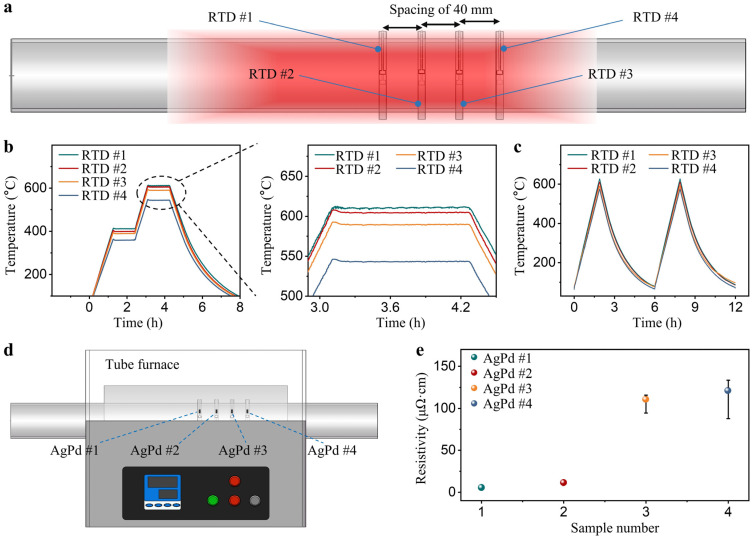
(**a**) Schematic diagram of the locations tested by the four Ag RTDs; (**b**) temperature curves of RTDs during simulated isothermal annealing process; (**c**) temperature curves of RTDs during simulated cyclic annealing process; (**d**) schematic diagram of the position of AgPd alloy thick films during sintering; (**e**) resistivity of AgPd alloy thick films after sintering.

Real-time monitoring of tube furnace operating conditions is essential for ensuring high-temperature safety, reliability, and controlling production quality. Multiple sets of temperature measuring rings were used for simultaneous temperature monitoring at different locations in the tube furnace to assess the corresponding sintering quality. An array of four Ag RTD temperature measurement rings was arranged sequentially from the tube furnace’s midpoint, with a 4 mm spacing between each (Figure 7a). The Ag RTDs were employed to monitor temperature conditions during the simulated isothermal annealing process, with test results presented in Figure 7b. At various annealing temperatures, the temperatures monitored by the rings at different positions decreased from the center to the periphery of the tube furnace. When the measured temperatures stabilized, the peak temperature range recorded by Ag RTD temperature rings at different positions ranged from 542 to 610 °C. Based on the measurement results, the average temperature gradient within a 12 mm range from the center of the tube furnace was calculated to be 5.7 °C/mm. Figure 7c shows the test curves of four RTDs during the simulated cyclic annealing process. The results indicated that the four RTDs exhibited good consistency over two cycles within the range of room temperature to 600 °C.

During the material synthesis process, variations in prepared samples often occur due to differences in their locations. The AgPd alloy slurry, due to its advantages of low TCR and high-temperature resistance, has been extensively utilized as a sensitive material for strain sensors and solder joint materials [44,45]. To investigate the impact of the temperature field within the tube furnace on the sintering quality of alloys, AgPd alloys underwent sintering at distinct locations corresponding to the temperature-measuring ring array (Figure 7d). Subsequently, the evaluation of their electrical resistivity was conducted. Figure 7e shows the resistivity of the AgPd alloys after isothermal annealing at four Ag RTD test locations. The corresponding resistivity statistical values distribute in the ranges of 5.2–121.1 μΩ·cm with a difference of more than 20-fold. This is because the mechanical properties, electrical characteristics, and grain size of the alloy are significantly influenced at various annealing temperatures [46]. The results above indicate that the temperature field within the tube furnace can be effectively monitored through the fabricated Ag RTD temperature measuring ring, enabling the health monitoring of the tube furnace and assessment of the sintering quality of specimens.

## 4. Conclusions

(1) The Ag RTD temperature measurement rings were fabricated on curved substrates using screen printing technology. The microstructure characterization results indicate that the prepared Ag film on the substrate was continuous, without any detachment or delamination phenomena;
(2) The fabricated Ag RTDs exhibit excellent electrical properties, including a TCR of 2854 ppm/°C across the range of 100–600 °C, a remarkable accuracy of 1.8% FS, and a high-temperature stability of 0.05%/h over a 6-h period at 600 °C. Furthermore, its response time under the same testing conditions was 0.5 min faster than that of commercial sheathed thermocouples;
(3) The developed Ag RTD temperature measuring ring was applied to real-time temperature monitoring of a tube furnace. The results show that four Ag RTDs with a spacing of 4 cm at different locations can distinguish different temperatures. Ag RTDs demonstrated the ability to accurately monitor temperatures for batch annealing processes. The AgPd alloys were sintered at the corresponding position, exhibiting different resistivity, demonstrating the accurate temperature monitoring capability of the Ag RTDs.

This work presents a new idea of using Ag film temperature sensors prepared on quartz ring substrates, which not only allow for precise position determination with tube furnaces but also allow for the most realistic temperature monitoring. Moreover, the use of this strategy is scalable, enabling array temperature monitoring of other pipe-like components in the future.

## Data Availability

Data are contained within the article.
